# Sensitive and specific fNIRS-based approach for awareness detection in disorders of consciousness: proof of principle in healthy adults

**DOI:** 10.1117/1.NPh.12.4.045001

**Published:** 2025-10-15

**Authors:** Lisa Bastian, Tim Näher, Anna Vorreuther, Michael Lührs, Amaia Benitez Andonegui, Pascal Fries, Lars Riecke, Bettina Sorger

**Affiliations:** a Maastricht University, Department of Cognitive Neuroscience, Maastricht, The Netherlands; b University of Tübingen, Institute of Medical Psychology and Behavioral Neurobiology, Tübingen, Germany; c Graduate Training Centre of Neuroscience, International Max Planck Research School, Tübingen, Germany; d Max Planck Institute for Biological Cybernetics, Tübingen, Germany; e Ernst Strüngmann Institute for Neuroscience in Cooperation with Max Planck Society, Frankfurt, Germany; f Radboud University Nijmegen, Donders Centre for Cognitive Neuroimaging, Nijmegen, The Netherlands; g Meta Reality Labs, Seattle, Washington, United States; h University of Stuttgart, Institute of Human Factors and Technology Management (IAT), Applied Neurocognitive Systems, Stuttgart, Germany; i Brain Innovation B.V., Research Department, Maastricht, The Netherlands; j NIH, MEG Core Facility National Institute of Mental Health, Bethesda, Maryland, United States

**Keywords:** coma, diagnosis, disorders of consciousness, functional near-infrared spectroscopy, machine learning

## Abstract

**Introduction:**

Disorders of consciousness (DoCs) are characterized by varying levels of arousal and awareness. Due to severe motor impairments often accompanying these conditions, differentiating between minimally conscious state (MCS) and unresponsive wakefulness syndrome (UWS) remains challenging. Accurate diagnosis, however, is critical for optimal treatment and prognosis. Functional near-infrared spectroscopy (fNIRS), due to its portability and noninvasiveness, holds promise for brain-based diagnostics, although current methods lack sufficient sensitivity and specificity.

**Methods:**

We introduce a highly sensitive, and specific fNIRS-based diagnostic approach tailored to the individual cognitive state of DoC patients. Nine healthy participants received auditory cues instructing them to either perform individually tailored motor-speech imagery or remain at rest. In separate runs, participants were instructed to be responsive to these cues to mimic MCS, or unresponsive, to mimic UWS. fNIRS–channel covariance matrices were classified for responsive and unresponsive states as either imagery or rest using a Riemannian-geometry-based approach.

**Results:**

Classification between responsive and unresponsive states achieved a sensitivity of 100% and a specificity of 89% across participants. Within the two states, imagery and rest were classified with 83,9% and 55,93% accuracy, respectively; the latter result, close to chance level, was expected in the unresponsive state.

**Conclusion:**

This individualized diagnostic approach may have the potential to significantly enhance diagnostic accuracy for DoCs. It provides a noninvasive, efficient, and objective assessment, potentially reducing the rate of misdiagnosis rates. The practicality and minimal technical requirements of fNIRS further support future clinical implementation.

## 1 Introduction

Disorders of consciousness (DoCs) are clinically defined by changes in arousal and/or awareness,^1^ classifying these conditions into three distinct states: (1) minimally conscious state (MCS) characterized by full arousal and minimal and inconsistent awareness, (2) unresponsive wakefulness syndrome (UWS) defined by full arousal but no awareness, and (3) coma, marked by an absence of both arousal and awareness.^2^^,^^3^ Although the behavioral diagnosis of coma is unequivocal, differentiating between MCS and UWS remains challenging.^4^ Patients in both states exhibit full arousal but may differ in their level of awareness, which is often difficult to detect. The most common diagnostic methods used in clinical practice, such as the Coma Recovery Scale-Revised^5^ and the Glasgow Coma Scale,^6^ exclusively rely on overt and controlled motor behavior. However, motor impairments, abnormal sleep-wake cycles, and uncontrolled movements in DoC patients create difficulties in the application of such behavioral assessments. This leads to ∼40% of MCS patients being inaccurately diagnosed as UWS.^7^ Accurate differentiation between UWS and MCS is critically important as MCS patients generally have a more favorable prognosis^8^ and are capable of communicating their needs through brain–computer interfacing (BCI).^9^ Furthermore, an incorrect diagnosis of UWS may lead to ethically challenging decisions, such as the termination of life-sustaining treatment, underscoring the urgent need for more accurate diagnostic tools.^10^^,^^11^

In light of these challenges, alternative, brain-based diagnostic approaches have been explored, combining instructions to perform an imagery task and noninvasive functional neuroimaging methods. These methods leverage mental rather than motor responses, with electroencephalography (EEG) and functional magnetic resonance imaging (fMRI) being the most extensively studied modalities^12^^–^^20^ (for a recent review, see Ref. 9). Despite their promise, both EEG and fMRI have notable limitations. EEG provides a high temporal resolution, yet it suffers from a substantial susceptibility to ocular and muscle-related artifacts. EEG measurements are also less sensitive in DoC patients with traumatic brain injury due to skull deformities.^20^ By contrast, fMRI offers excellent spatial resolution through measuring blood-oxygen-level-dependent (BOLD) signals but lacks bedside applicability due to its stationary nature. Functional near-infrared spectroscopy (fNIRS) emerged as a promising alternative modality for detecting awareness.^21^ Most importantly, fNIRS simultaneously measures changes of both oxygenated (HbO) and deoxygenated (HbR) hemoglobin, which can provide complementary sources of information^22^ and therewith potentially more diagnostic certainty. FNIRS is affordable, practical, and quieter compared with fMRI and has reduced susceptibility to artifacts compared with EEG.^23^ However, it is limited by potential signal interference from hair density and skin pigmentation;^24^ for a detailed evaluation, see Ref. 23.

Despite its significant and unique clinical potential, fNIRS-based awareness detection in DoC patients has been explored in only a small number of studies. Existing research has identified residual awareness through resting-state functional connectivity,^25^^,^^26^ passive paradigms,^27^^,^^28^ or active paradigms.^28^^–^^32^ Active paradigms generally yield more robust and reliable results^21^ and can subsequently be employed for BCI-mediated communication.^33^ Using an active paradigm in MCS/UWS patients, Abdalmalak et al.^29^ achieved 79% to 83% diagnostic sensitivity and 58% to 71% specificity, highlighting its great promise for DoC diagnosis.^21^ However, we believe that experimental designs and procedures need to and can be further improved.

A potential area for improvement is the implementation of the mental imagery tasks. Existing active paradigms rely on predefined imagery tasks with long encoding times (7 to 30 s), which may impose unnecessary cognitive demands and overlook individual differences in patient capabilities due to their unique patterns of brain damage. To overcome this, we here suggest and test an individualized imagery task, composed of a motor and speech component, with a short encoding time of only 2 s. Importantly, patients could choose the exact motor- and speech-imagery strategy themselves depending on their unique mental capabilities. Another potential limitation of existing paradigms is the analysis method. All the studies mentioned above relied on a canonical hemodynamic response function (HRF) that assumes a uniform response shape. In addition, most of the studies did not consider multichannel activation patterns which, by definition, provide more potentially meaningful information than single-channel or channel-average analyses. To address this, we used a more sensitive covariance-based classification approach that considers co-activation patterns between fNIRS channels without relying on a predefined HRF shape. By reducing cognitive demands and employing a more sensitive analysis approach, our paradigm has the potential to improve diagnostic accuracy and efficiency in clinical practice.

The present study validated the novel diagnostic approach in healthy adults. Per trial, participants received auditory task cues instructing them to either perform an individualized motor-speech IMAGERY task for 2 s or a NO-IMAGERY resting task for 2 s. Furthermore, in separate experimental runs, participants were instructed to either be RESPONSIVE to the trial-wise task cues, thereby mimicking MCS, or to be UNRESPONSIVE, thereby mimicking UWS. We classified IMAGERY versus NO-IMAGERY conditions in both the RESPONSIVE and UNRESPONSIVE states. We hypothesized that classification accuracy significantly exceeds chance level in the RESPONSIVE but not in the UNRESPONSIVE state. Using a Riemannian-based classification approach, our diagnostic tool achieved high sensitivity and specificity, demonstrating potential for improvements in the differential diagnosis of DoC patients.

## 2 Methods

### 2.1 Participants

We recruited 14 healthy adults with an age of 27.5 ± 11.4 years (mean ± SD) and normal hearing, including external volunteers, and students and staff members of the Faculty of Psychology and Neuroscience at Maastricht University. Five participants were subsequently excluded from the analysis, for the following reasons: Two participants were excluded due to data corruption, one participant had insufficient fNIRS signal quality due to physical attributes (i.e., hair thickness and skin pigmentation), another participant fell asleep during the task after extensive physical exercise before the fNIRS session, and the fifth participant reported claustrophobic symptoms in the test cabin (see Table 1 for participant characteristics). Participants gave written informed consent and received financial compensation for their participation. The local ethics committee approved the experiment (Ethics Review Committee Psychology and Neuroscience [ERCPN]).

**Table 1:** Participant demographics.

Participant	Sex	Age (years)	Handedness	Cap size used (cm)
P01	F	21	Right	58
P02	F	47	Right	56
P03	F	23	Right	56
P04	F	25	Right	56
P05	F	23	Right	58
P06	F	20	Right	58
P07	F	22	Right	56
P08	F	27	Right	58
P09	M	22	Right	58

F = female; M = male.

### 2.2 General Procedure

**Fig. 1:**
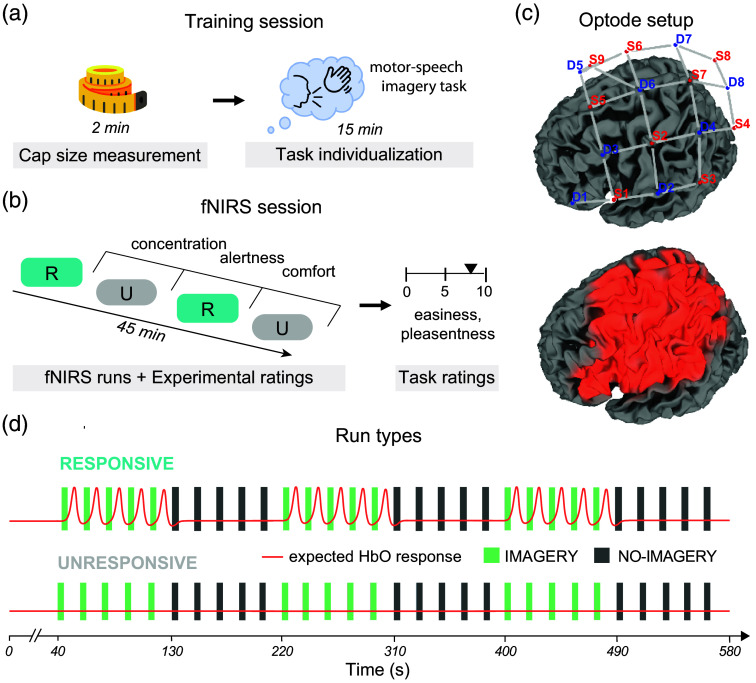
Experimental procedure. (a) Prior to fNIRS-data collection, participants completed a training session. This session included cap-size determination and a 15-min task-individualization procedure in which subjects could define their own motor–speech imagery strategy for the task. (b) The fNIRS session took ∼1  h and consisted of four (two RESPONSIVE and two UNRESPONSIVE) fNIRS runs lasting 09:40 min each. The runs alternated between RESPONSIVE and UNRESPONSIVE. Half of the participants started with RESPONSIVE, and the other half with UNRESPONSIVE. In between each run, participants rated their level of experienced alertness, concentration, and comfort. After data collection, participants indicated easiness and pleasantness of the mental–imagery task and the resting task. (c) The optode setup included eight detectors and nine sources over the left hemisphere, covering both motor and speech areas. The brain regions marked in orange show the light-intensity map and thereby the regions covered by the fNIRS montage. We also included a short-distance channel (S9-D5). (d), Schematic representation of the blocked event-related design for RESPONSIVE and UNRESPONSIVE runs. IMAGERY and NO-IMAGERY trials were performed in blocks of five. Each trial consisted of a 1-s auditory cue of high or low frequency and a 2-s task time. Between trials, there were 15-s resting periods. The allocation of the two sound frequencies (high and low) to prompt the two task conditions (IMAGERY or NO-IMAGERY) was balanced across the four fNIRS runs.

The experiment consisted of a training session [Fig. 1(a)] and the fNIRS session. During the training session, participants were familiarized with the mental-imagery task they had to perform in the IMAGERY condition within the RESPONSIVE state. The task consisted of 2-s-long, combined motor–speech imagery. As the encoding time of 2 s is very short, and some DoC patients may have impairments in either the motor or speech domain, we decided to combine motor and speech components to increase the likelihood of evoking a robust hemodynamic response in as many participants as possible.^34^ To allow for a user-centered approach, participants could choose the specific strategy to perform the task. To guide the participant’s choice of strategy, they were asked to think of how they would naturally (within ∼2  s) draw attention to themselves if they were in the situation of a DoC patient by imagining to, e.g., waving their hands/knocking and shouting “Hello, I am here!”. The training session ended with measuring the head size and determining the respective fNIRS-cap size.

One to two days after the training session, participants performed an fNIRS session [Fig. 1(b)]. At the beginning of the session, participants were seated comfortably in a noise-dimmed cabin. The fNIRS cap was placed on the participant’s head, and all optodes were adjusted to ensure orthogonality to the scalp and proper optode-scalp contact. Participants were instructed to sit still, close their eyes, and avoid any movements during the recordings. The fNIRS session lasted for ∼1  h, including participant preparation and data-acquisition [Fig. 1(b)].

### 2.3 Imagery Paradigm

During the fNIRS session, participants performed four runs, each lasting 09:40 min. Each run started with a 40-s baseline period, after which participants performed six blocks of five trials each, alternating between IMAGERY and NO-IMAGERY blocks. The order of blocks was counterbalanced across runs. Each trial lasted 3 s, including an auditory cue of high (1000 Hz) or low frequency (300 Hz) of 1 s, a task period of 2 s, followed by a resting period of 15 s. Out of the four runs, two runs were RESPONSIVE, whereas the other two were UNRESPONSIVE [Fig. 1(b)]. RESPONSIVE and UNRESPONSIVE runs were performed in alternation; half of the participants started with RESPONSIVE and the other half with UNRESPONSIVE. We used two separate RESPONSIVE and UNRESPONSIVE runs to account for within-session variability of fNIRS data quality and alertness, i.e., to reduce potential fatigue and attentional drift effects within a single long block. The RESPONSIVE state was used to simulate an MCS patient, who is able to follow a command by willful modulation of brain activity [Fig. 1(d)]. For IMAGERY trials, participants performed the mental task in response to one type of auditory cue (high- or low-frequency sound). For NO-IMAGERY trials, they did not perform the mental-imagery task indicated by the other sound. The UNRESPONSIVE state was used to simulate a UWS patient, who cannot show mental command following. Here, participants were instructed to never perform the mental–imagery task, neither in IMAGERY nor in NO-IMAGERY trials, which were again indicated by two different sound frequencies [Fig. 1(d)]. The order of auditory cues was counterbalanced across participants for IMAGERY and NO-IMAGERY trials.

### 2.4 Data Acquisition

A NIRScout-816 system (NIRx Medizintechnik GmbH, Berlin, Germany) was used to measure the hemodynamic response, using nine source and eight detector optodes [Fig. 1(c)]. Sources were positioned according to the international 10-20 EEG system on FC5 (1), C3 (2), CP5 (3), P3 (4), FC1(5), Cz (6), CP1 (7), Pz (8), and at the midsagittal dimension between Cz and FCz (9). Detectors were positioned on F5 (1), C5 (2), FC3 (3), CP3 (4), FCz (5), C1 (6), CPz (7), and P1 (8). Source 9 and detector 5 formed a short-distance channel with a 1-cm optode distance to obtain information on physiological noise (i.e., Mayer waves [∼0.1  Hz]) within the superficial layers, excluding brain tissue and affecting specifically HbO time courses.^35^ Information obtained from this channel was used later to regress out systemic noise from the 3-cm normal-distance channels. The placement of the entire cap took on average ∼5  min. Placed optodes covered a region above the left-hemispheric fronto-parietal cortex where we expected motor and speech task-related modulation of brain activation. Data were recorded using NIRStar 15.2 (NIRx Medizintechnik GmbH, Berlin, Germany) at 6.94 Hz.

### 2.5 Subjective Ratings

After each run, participants rated their level of experienced concentration, alertness, and comfort according to a Likert scale ranging from 1 (extremely unconcentrated/tired/ uncomfortable) to 10 (extremely concentrated/alert/comfortable). Upon completion of the experiment, participants rated the general easiness and pleasantness of the mental imagery task and resting task on a Likert scale ranging from 1 (extremely difficult/unpleasant) to 10 (extremely easy/pleasant).

### 2.6 Data Analysis

#### 2.6.1 Data preprocessing

The fNIRS data were preprocessed with the MNE library^36^ in Python 3.10. Initially, the raw data were visually inspected during data acquisition for salient signal increases after trial onset. We again inspected the data offline prior to the analysis using pulse oscillations as a marker for signal quality. Based on the inspection, no trials were removed. Then, the raw signals were converted into optical densities. After this transformation, we used temporal derivative distribution repair to rectify baseline shifts and eliminate spike artifacts. Short distance-channel regression was applied using the MNE implementation (based on Refs. 37–39) to remove Mayer waves and other extracerebral noise. Next, optical densities were converted to hemoglobin-concentration changes by applying the modified Beer–Lambert law with a partial path length factor of 6. The data were then bandpass-filtered using a second-order Butterworth filter (low-pass and high-pass cut-offs at 0.1 and 0.4 Hz, respectively) and segmented into epochs. Each epoch was defined starting from the auditory cue onset and extending to 12s post-onset. Linear trends were removed from each epoch.

### 2.7 Classification

Using a novel approach, the analysis aimed to classify participants as RESPONSIVE, mimicking MCS, or UNRESPONSIVE, mimicking UWS. Typically, classifiers are trained on a dedicated training set and then used to make predictions for new, unseen data. By contrast, our approach utilized the successful discrimination between IMAGERY and NO-IMAGERY as an indicator of the participant’s compliance with the task instructions. The logic of this approach is as follows: If DoC patients can adhere to instructions (i.e., they are, for example, in an MCS), they should be able to modulate their brain activity by performing a mental–imagery task. When trained on such data, this modulation implies that a classifier can distinguish between IMAGERY (when the patient engages in the imagery task) and NO-IMAGERY (when the patient continues to rest) trials. Conversely, if a DoC patient is unresponsive (i.e., is in UWS) and thus unable to follow the task instruction, their brain activity should not exhibit such modulation. Consequently, in such cases, a classifier should fail to differentiate between IMAGERY and NO-IMAGERY trials, resulting in classification accuracies close to chance level. Each participant mimicked both an MCS and a UWS patient by being RESPONSIVE in two runs and UNRESPONSIVE in the other two runs.

**Fig. 2:**
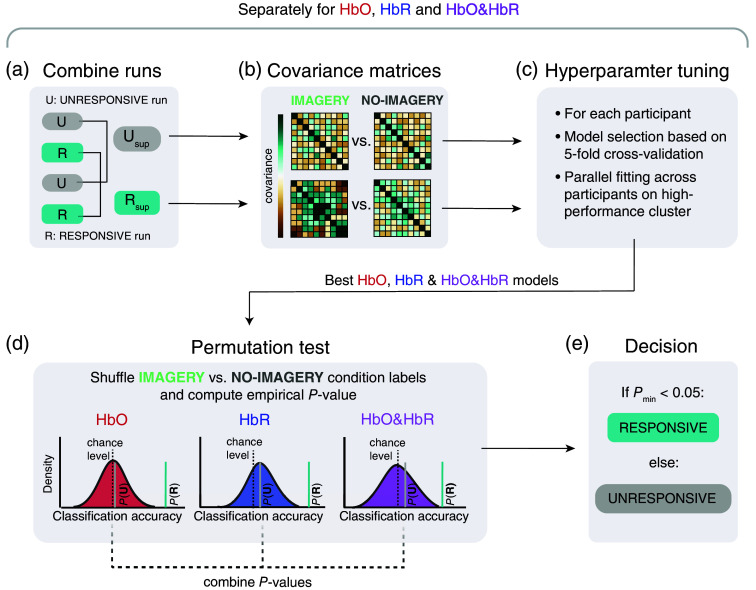
Covariance-based classification analysis. (a) The four individual runs were combined into “super runs” for subsequent analyses. (b) Special-form covariance matrices were computed based on all channels and condition-specific trials per participant. (c) For each participant, we conducted a small hyperparameter search to identify the best model, separately for HbO, HbR, and HbO & HbR. We determined the best model based on a fivefold stratified cross-validation accuracy score. The procedure was conducted in parallel for all participants on a high-performance cluster. (d) The best models’ final classification accuracies were tested against empirical null hypotheses with a permutation test (N=250 permutations). (e) In a final step, we classified the participant as RESPONSIVE if any P-values were below the threshold of 0.05, or else as UNRESPONSIVE.

We combined trials from corresponding runs for classification, resulting in 60 trials per responsiveness condition in each participant [Fig. 2(a)]. We then computed the channel covariance matrices for the two combined runs (cf. Sec. 2.8). We conducted a hyperparameter search to determine optimal classifier model parameters (Fuertinger, Shapcott, and Schmiedt, 2023).^40^ For each hyperparameter set, we obtained classification accuracies by a stratified fivefold cross-validation. Based on these accuracies, we selected the hyperparameter set that yielded the highest classification accuracy and retrained the respective model [Fig. 2(c)]. All analyses were performed separately for HbO, HbR, and combined (i.e., HbO & HbR) data. Note that we used full covariance matrices as features for our classifier, resulting in the dimensionality of the combined (HbO & HbR) being larger than that of just HbO or HbR. The dimensionality of the manifold that is used for classification is given by the number of independent elements in the covariance matrix:

$$dim(M)=\frac{n(+1)}{2},$$

where M is the manifold of n×n symmetric positive definite matrices (e.g., covariance matrices). In addition, the combined matrix thus contains the covariances between HbO & HbR.

### 2.8 Covariance Estimation and Classifier

We used a classification based on the covariance matrices computed over all channels [Fig. 2(b)]. Applying covariance-based classification is a recent development in the fNIRS data analysis field that has shown promising results as a sensitive method for detecting small differences in mental brain states.^41^ Using the covariances between channel pairs has the advantage that no prior channel selection is required and no canonical HRF needs to be assumed. Here, we estimated a special form of the covariance matrix that is specifically useful for the analysis of event-related potentials.^42^^,^^43^ Briefly, a prototyped response P is obtained by computing the average signal separately over IMAGERY and NO-IMAGERY trials in the training set:

$$P=\frac{1}{m}\sum_{i=1}^{m}X_{i},$$

where m is the number of respective trials, and $X_{i}$ represents the time series data of trial i. The prototyped response P is then concatenated with $X_{i}$

$${\overset{˜}{X}}_{i}=[\begin{array}{c} P\\ X_{i} \end{array}].$$

Conceptually, this is equivalent to computing the average response over trials for each channel and treating these averages as new channels. The resulting concatenated trials ${\overset{˜}{X}}_{i}$ are then used for covariance estimation. Adding the average, event-related responses of IMAGERY and NO-IMAGERY allows us to take the spatial structure of the signal into account by including the covariances of the individual trials with the prototypes’ responses represented by P. Note that for HbO & HbR, each channel was represented by both HbO & HbR data, effectively doubling the channel count.

To reduce the dimensionality of covariance matrices and speed up computation during model fitting, we applied the xDAWN algorithm, a spatial filtering technique commonly used in EEG analyses, to boost the signal-to-noise ratio in event-related potentials.^44^^,^^45^ Before concatenation, the xDAWN filters were applied to both the prototypes P and the individual trial data $X_{i}$. For the final covariance estimation, we used the regularized oracle approximating shrinkage (OAS) method.^46^ Regularized covariance estimation provides more numerical stability when covariances are estimated on small samples as is the case here. In addition, we compared different regularizations and found that OAS demonstrated the highest numerical class distinctiveness in differentiating IMAGERY and NO-IMAGERY trials in the RESPONSIVE condition (see Sec. 2.9 for more details).

Covariance matrices computed from linearly independent data are inherently symmetric and positive definite, meaning they belong to the space of symmetric positive definite (SPD) matrices. This space forms a Riemannian manifold—a curved space that locally approximates flat Euclidean geometry through its tangent spaces. As each point on the manifold has an associated tangent space that “touches” the manifold, we can perform well-defined geometric operations in these local coordinates. In our approach, we exploit this structure by projecting the spatially filtered and regularized covariance matrices onto their corresponding tangent spaces. This projection maps each covariance matrix onto a Euclidean vector with dimensionality that depends on the number of spatially filtered channels, effectively preserving the local structure of the original manifold. We employed a Riemannian metric both for estimating the reference matrix and for performing the tangent-space mapping, following the methodology described by Barachant et al.^47^ The reference matrix, typically computed as the Riemannian mean of the covariance matrices, serves as the common anchor point from which the tangent-space mapping is derived, preserving the intrinsic geometry of the SPD manifold. Finally, these Euclidean-transformed vectors were classified using binary logistic regression to distinguish between IMAGERY and NO-IMAGERY. The hyperparameter search for this pipeline included the number of spatial filters for the xDAWN algorithm (2, 4, or 6 filters) and the regularization term C for the logistic regression (1, 10, 100, or 1000).

### 2.9 Class Distinctiveness

We compared the class distinctiveness on the manifold of covariance matrices for the two conditions IMAGERY versus NO-IMAGERY. We followed the definition from Ref. 48

$$\text{classDis}(A,B)=\frac{d({\overset{¯}{X}}^{A},{\overset{¯}{X}}^{B})}{\frac{1}{2}(\sigma_{X_{A}}+\sigma_{X_{B}})},$$

where ${\overset{¯}{X}}^{A}$ and ${\overset{¯}{X}}^{B}$ are the centers of IMAGERY and NO-IMAGERY, i.e., the mean of matrices from each class, $\sigma_{X_{A}}$ and $\sigma_{X_{B}}$ are the class dispersions, i.e., the mean of distances between matrices from class K and their center of class

$$\sigma_{X_{K}}^{p}=\frac{1}{m}\overset{m}{\underset{i=1}{\sum }}d(X_{i},{\overset{¯}{X}}^{K}{)}^{p}.$$

We found that OAS estimation of covariances resulted in the highest numerical class distinctiveness. OAS regularization was then chosen for all subsequent covariance estimations.

### 2.10 Thresholding and Permutation Tests

To classify each participant dataset as RESPONSIVE or UNRESPONSIVE, we used permutation tests,^49^ which involved randomly shuffling the IMAGERY and NO-IMAGERY trial labels 250 times to obtain a null distribution and comparing it to the observed cross-validation accuracy to obtain an empirical P-value. We applied this test independently to HbO, HbR, and the combined (HbO&HbR) data [Fig. 2(d)]. To obtain a final prediction for each state, we took the minimum P-value of the three separate analyses (HbO, HbR, and HbO & HbR). A dataset was classified as RESPONSIVE if its minimum P-values was below the threshold of a=0.05 [Fig. 2(e)]. Such a result would suggest that the observed cross-validation accuracy significantly exceeded what would be expected by chance, indicating the condition as RESPONSIVE.

### 2.11 Further Statistical Analyses

For all statistical tests, we set a significance threshold of a<0.05, corrected for multiple comparisons. The statistical analyses described in this section were conducted in R Studio (Version 2021.09.1). Average classification accuracies are displayed as mean ± 1 standard deviation. Nonparametric Wilcoxon signed-rank tests were used to assess the difference between RESPONSIVE and UNRESPONSIVE for HbO & HbR, HbO, and HbR and task ratings. Spearman correlations were computed between, on the one hand, overall classification accuracies of RESPONSIVE/UNRESPONSIVE states and, on the other hand, either task ratings or performance ratings (i.e., alertness, concentration, and comfort). To assess the interaction effect of responsiveness (RESPONSIVE versus UNRESPONSIVE) and time (RUN 1-4) on performance ratings, we computed a two-way ANOVA. Effect sizes for the F-statistics are reported by eta-squared.

## 3 Results

### 3.1 Covariance-Based Classification Accuracy

**Fig. 3:**
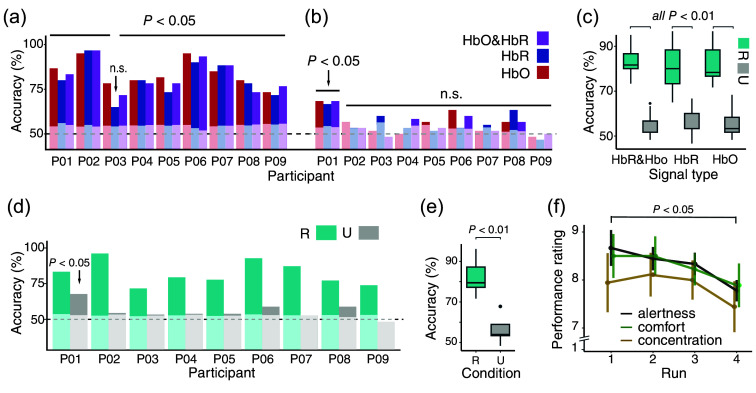
Classification accuracies and performance ratings. (a) Individual participants’ classification accuracies for the RESPONSIVE state for HbO, HbR, and HbO & HbR separately. White shadings indicate individual empirical chance levels. All runs showed significant classification accuracies above the empirical chance level (P<0.05), except the HbO data for P03. (b) Same as A but for the UNRESPONSIVE state. For all participants, except P01 (P<0.05), accuracies did not differ significantly from the empirical chance level. (c) Mean accuracy over all participants, separately for HbO (R: 80.37%±9.35% N: 55.92%±5.89%), HbR (82.22%±8.31%, U: 55.56%±5.78%), and HbO & HbR (R: 83.89%±6.93%, U: 55.93%±6.14%) for the RESPONSIVE and UNRESPONSIVE states (all P<0.01). During the RESPONSIVE state, classification accuracy was higher compared with the UNRESPONSIVE state for HbO, HbR, and HbO & HbR (P<0.01). (d) Average accuracy based on HbO, HbR, and HbO & HbR results. RESPONSIVE data were classified correctly based on $P_{\min\;}<0.05$. For the UNRESPONSIVE condition, no participant except P01 surpassed the chance level based on $P_{\min}<0.05$. (e) Mean group accuracies combining HbO, HbR, and HbO & HbR (R: 82.16%±8.40%, N: 55.80%±5.51%; P<0.01). (f) Participants’ mean alertness 8.47±0.83, comfort 8.95±0.97, and concentration 8.46±0.92 ratings throughout the fNIRS runs. Alertness decreased significantly from run 1 to run 4 (Wilcoxon V = 30, P<0.05).

When participants followed task instructions (during RESPONSIVE states), we were able to classify IMAGERY versus NO-IMAGERY trials above chance level. The average classification accuracy was the highest for HbO & HbR (83.89%±6.93%), followed by HbR (82.22%±8.31%), and HbO 80.37%±9.35%; Fig. 3(c)]. When classification was based on HbO, accuracy exceeded the chance level for eight out of nine participants (all P≤0.01) and trended for participant P03 with an accuracy of 65% (P=0.08). When HbR or HbO & HbR were used, all participants were classified correctly above chance level (all P≤0.01) in the RESPONSIVE state [Fig. 3(a)].

As expected, in the UNRESPONSIVE state, when the classifier did not receive two meaningfully distinct classes, average classification accuracy was around chance level for HbO (55.92%±5.89%), HbR (55.56%±5.78%), as well as for HbO & HbR [55.93%±6.14%, Fig. 3(c)]. Eight out of nine participants were classified at chance level for all three hemodynamic signals. Only one participant (P01) was inaccurately classified above chance level for HbO (66.67%, P=0.02), HbR (68.33%, P=0.02), and HbO & HbR [68.33%, P=0.01, Fig. 3(b)].

We observed that the fNIRS-signal amplitudes at a single-trial level were generally higher in the RESPONSIVE than the UNRESPONSIVE state for both HbO & HbR in IMAGERY trials, showing clear task-evoked brain responses only in the RESPONSIVE state (see example participant in Fig. S1 in the Supplementary Material). As expected, classification accuracy based on the covariance matrices of the fNIRS signals across channels was higher in the RESPONSIVE compared with the UNRESPONSIVE state for HbO&HbR (Wilcoxon V = 45, P<0.01), for HbO (V = 45, P<0.01), and HbR [V=45, P<0.01; Fig. 3(c)]. Similar results were obtained when averaging over HbO, HbR, and HbO & HbR for each participant [Fig. 3(d)]. The average classification accuracy was 82.16% (±8.40%) in the RESPONSIVE state, with a minimum of 71.67% (P03) and a maximum of 96.11% (P02). By contrast, the average classification accuracy for the UNRESPONSIVE state was around chance level at 55.80% (±5.51%). On average, classification accuracy in the RESPONSIVE state was higher than during the UNRESPONSIVE state [V=43, P<0.01, Fig. 3(e)].

To make a final judgment whether a participant was responsive or not, we took the minimum P-value obtained for HbO, HbR, and HbO&HbR. Based on this, we could correctly classify all participants as responsive, yielding 100% sensitivity (9/9 participants). For the UNRESPONSIVE state, we obtained one false positive classification (P01), resulting in a specificity of 89% (8/9 participants). These results demonstrate that our covariance-based classification approach is highly sensitive (100%) and specific (89%) in detecting responsiveness to the instruction to perform a mental–imagery paradigm.

### 3.2 User Ratings and Classification Accuracy

To better assess the user experience of our paradigm, we acquired task ratings and performance ratings. Overall, the mental-imagery task was rated as easy (7.93±1.24) and pleasant (8.81±1.26) to perform. The mental–imagery task performance in the RESPONSIVE state was rated as being easier (Wilcoxon V=31, P=0.04) but not more pleasant (Wilcoxon V = 21.5, P=0.33) than being passive in the UNRESPONSIVE state. Both easiness and pleasantness of being RESPONSIVE or being UNRESPONSIVE were not associated with classification accuracies (all P≥0.35).

Furthermore, comfort, alertness, and concentration ratings during task performance were overall high (comfort: 8.95±0.97, alertness: 8.47±0.83, concentration: 8.46±0.92) but followed a similar downward trend across runs [Fig. 3(f)]. Specifically, alertness dropped significantly between runs 1 and 4 (Wilcoxon V = 30, P<0.05). The small decrease in alertness was not correlated with a possible decrease in classification accuracy in the RESPONSIVE (*ρ* = 0.28, P=0.46) or UNRESPONSIVE state (ρ=0.13, P=0.74). All three ratings were not significantly different between RESPONSIVE and UNRESPONSIVE across all runs (comfort: F(3,24)=0.64, P=0.60, $\eta^{2}=0.03$; alertness: F(3,24)=2.26, P=0.10, $\eta^{2}=0.02$; concentration: F(3,24)=0.62, P=0.60, $\eta^{2}<0.01$).

## 4 Discussion

This proof-of-principle study successfully implemented an fNIRS-based diagnostic tool for DoC patients in healthy adults. By employing command following through personalized mental imagery as a surrogate for MCS and UWS conditions, we demonstrated the potential of fNIRS for the differential diagnosis of DoC patients. Notably, the channel covariance-based classification achieved 100% sensitivity (9/9 participants) and 89% specificity (8/9 participants) in detecting participants’ responsiveness, underscoring the potential of this highly sensitive and specific approach for clinical application.

### 4.1 Sensitive and Specific Diagnostic Tool for Bedside Assessment

The development of a sensitive and specific bedside diagnostic tool is essential to advance the clinical assessment of DoC. In this study, we achieved correct classifications across almost all participants (except one). To the best of our knowledge, we are the first to show a 100% sensitivity in detecting volitional command-driven brain activity in healthy adults. In a recent study, Kazazian et al.^30^ reported a 62.5% sensitivity for a classical motor imagery task, and only 33.33% for spatial navigation. Our sensitivity is also higher than that reported in previous fMRI work (e.g., Edlow et al.^2^—68.5%, Fernandez-Espejo et al.^50^—78.5%). In addition, we achieved this sensitivity using a paradigm that requires only 20 min (60 trials per condition). This outcome suggests that our approach is highly effective with a relatively small trial number.

For bedside diagnostics, it is crucial to balance the number of trials needed for reliable classification with the cognitive demands placed on patients. To address this, we assessed participants’ subjective ratings of concentration, comfort, and alertness, which remained high and stable across four experimental runs. Although there was a slight but significant decrease in alertness from the first to the last run, this effect is likely attributable to the experimental setup. Our participants completed four runs to simulate both MCS and UWS conditions, which would not be necessary in clinical settings, where patients would be either responsive or unresponsive. Therefore, they would only need to complete half of the trials, reducing both task-switching effects and the overall test duration. In addition, our testing environment—a small, dark, isolated cabin designed to minimize external noise and enhance signal quality—likely contributed to a decline in alertness over time.

Another central challenge in diagnosing DoC patients lies in the heterogeneity of individual brain-injury etiologies. Traditional analyses of hemodynamic responses often rely on a canonical HRF that assumes a uniform response shape. By contrast, our model-free covariance matrix approach does not depend on a predefined HRF, accommodating individual variability in hemodynamic response shapes. This flexibility is particularly valuable in the context of DoC, where patients exhibit diverse brain-injury patterns that affect brain response profiles.^51^

FNIRS measures two hemodynamic signals, namely, HbO & HbR. We used HbO, HbR, and HbO & HbR channel covariances to separately classify datasets as RESPONSIVE or UNRESPONSIVE. In line with previous support vector machine classification results in DoC,^52^ we achieved the highest classification accuracy for the combined HbO & HbR data on a group level. This underscores the importance of leveraging both signals and their interactions to differentiate levels of mental engagement. Nevertheless, the combined HbO & HbR unequivocally yielded the maximum classification accuracy for only one individual participant, whereas either HbO (5/9) or HbR (3/9) alone yielded the best results for most participants on an individual level. Due to these individual differences in response characteristics, we based our responsiveness diagnosis on the contributions of HbO, HbR, and HbO & HbR, resulting in more sensitive predictions.

The one instance of misclassification (P01) in the UNRESPONSIVE condition raises important implications. A classifier trained on the data of this participant was able to significantly differentiate IMAGERY from NO-IMAGERY trials (HbO: P=0.02; HbR: P=0.02; HbO&HbR: P=0.01), suggesting command following contrary to task instructions. This could indicate that the participant did not act unresponsively. Indeed, remaining at rest during the UNRESPONSIVE condition was experienced as more difficult by our participants than being active during the RESPONSIVE condition. However, in contrast to our healthy controls, UWS patients who are truly unresponsive do not have to actively inhibit a response. Therefore, this misclassification should not arise in clinical diagnosis. Nevertheless, misclassification in general emphasizes potential limitations of relying solely on fNIRS data and underscores the value of integrating our paradigm into a broader diagnostic framework to minimize false diagnoses in DoC patients.

### 4.2 Novel and Practical Classification Approach for fNIRS Data

To the best of our knowledge, we are the first to show an fNIRS-based proof of principle in healthy participants of the use of Riemannian geometry for brain-based classification with direct clinical relevance. Covariance matrices are symmetric positive definite matrices and can be treated as points on a Riemannian manifold. We exploited this characteristic by performing classification in the tangent space of the channel covariance matrices using logistic regression. This approach can be computationally more efficient than conventional classification approaches because each trial is represented by a single matrix rather than multichannel HbO & HbR data that contain an additional temporal dimension. We further reduced the dimensionality of covariance matrices before classification by applying a spatial filter.^44^ This approach demonstrates new opportunities to apply multivariate classification of fNIRS data in applied settings.

Given time and monetary constraints in clinical practice,^53^^,^^54^ we aimed at making results readily available within <5  min after data acquisition while still maintaining high accuracy. This was specifically achieved by reducing covariance dimensionality, a small number of permutations (250), and a small parameter space during the hyperparameter search. The hyperparameter search was conducted to obtain a good fit for each classifier and was carried out for HbO, HbR, and HbO & HbR, and each participant separately. The small parameter set reflected a trade-off between individually tailored classifiers and feasible fitting times (<3  min). Noteworthy is that these durations may vary depending on the computational performance of the machine.

In most machine learning applications, classifiers are trained on a separate training dataset and evaluated on a dedicated test dataset to assess generalizability. However, this split is unnecessary in our approach because our objective is not to evaluate classifier generalization but rather to use the successful training of classifiers as evidence of meaningful structure within the run. This structure, in turn, serves as an indicator for distinguishing between responsiveness and unresponsiveness during command-following tasks. To assess performance, we use cross-validation accuracy, which requires fewer trials than a traditional train–test split. This reduces the burden on patients with DoC and enables faster, clinically actionable results. In addition, training individual models for each diagnostic session aligns with emerging trends in personalized medicine and diagnostics. In the context of DoC, using pre-trained models on large populations is likely not ideal due to the high heterogeneity of patients, symptoms, and impairments. For such models to generalize effectively, they must account for substantial variability in fNIRS equipment (e.g., optode placements and preparation) and diverse clinical profiles. Our approach avoids this generalization challenge entirely. Here, classification is performed within specific sessions and does not need to generalize across different settings and patients. Instead, we demonstrate that Riemannian-based classification performs well with a small number of examples^41^ and that retraining the model for each diagnostic run is both feasible and highly suitable for bedside clinical applications.

### 4.3 Benefits of a User-Friendly Mental-Imagery Task

Our paradigm is centered on a concise, user-friendly mental-imagery task, combining motor and speech imagery in 2-s trials. This design prioritizes minimal encoding time to reduce mental load and fatigue, potentially enhancing diagnostic accuracy in clinical applications. Previous studies using mental–imagery tasks employed significantly longer encoding durations, ranging from 12 s up to 30 s.^31^^,^^32^^,^^55^ Despite the presumed advantage of these longer tasks for producing a robust hemodynamic response (HR), the results have been mixed. Thus, our approach aimed to boost a robust HR with 2-s mental imagery. In a recent fNIRS-based BCI study, Vorreuther et al.^33^ successfully implemented this very short 2-s encoding time in a binary communication paradigm in healthy adults. Further research is required to confirm the suitability of this 2-s interval for DoC patients.

Mental-imagery tasks used in DoC diagnostics have varied in effectiveness, with motor-related tasks generally outperforming cognitively complex paradigms such as mental arithmetic or spatial navigation.^30^^,^^32^^,^^55^ We implemented an individualized combined motor–speech imagery task, which we think is tailored for DoC patients for two reasons:

First, the task takes advantage of two modalities (i.e., motor and speech) that have both been shown to elicit robust HR in premotor/supplementary motor areas and Wernicke’s area.^33^ As patients often have unique brain damage, capturing the activation of multiple cortical regions may increase the chance of observing task-evoked responses, even though some of the regions may not be fully functional anymore. Note that our optode layout was intentionally limited to the left hemisphere, primarily to ensure swift and practical placement, which is essential for potential clinical use to reduce the burden on caregivers and patients. This left-lateralized montage may be optimal for capturing activation patterns related to speech and right-sided motor imagery—the dominant side for all our participants (see Table 1)—but not for left-handed individuals or those with atypical hemispheric dominance. Therefore, future studies may examine bilateral or hemisphere-tailored optode placements, potentially guided by handedness, to ensure optimal signal quality and generalizability.

Second, our mental-imagery task was individualized for the participants. Each participant was free to choose the specific strategy for the mental–imagery task they felt most comfortable with. In contrast to active paradigms currently used for DoC diagnosis,^28^^–^^32^^,^^55^ we did not give explicit task instructions but merely guided participants to choose a combined, motor–speech strategy with which they would try to get noticed if they were in the situation of an MCS patient. Giving this degree of freedom to patients allows adjusting the task to their mental capacities and may consequently increase the chance of obtaining a robust HR. The user-friendliness of the task is also reflected by consistently high ratings of easiness and pleasantness of our participants.

### 4.4 Limitations and Future Directions

The present study has some limitations that should be acknowledged. Overall, the sample size was relatively small (N=9). Although this limits the generalizability of group-level findings and reduces statistical power to detect smaller or more nuanced effects, the primary aim of this study was not to establish population-level inferences but to demonstrate the feasibility of our individualized diagnostic paradigm on a single-participant level. Our approach relies on within-subject classification of mental imagery versus rest to assess responsiveness, which aligns with the needs of clinical diagnostics that require reliable detection at the level of individual patients. Indeed, each participant served as their own control, and classification accuracy was assessed via permutation-based significance testing at the individual level. Nevertheless, replication in a larger and more demographically diverse sample will be essential for future validation, particularly to evaluate classifier robustness across different physiological and cognitive profiles. A multicenter study or replication in a different lab with expanded recruitment would be a valuable next step toward clinical translation.

In addition to the sample size, our present sample has very limited diversity, including only one male participant and mostly young adults with lighter skin. To assess the generalizability of our paradigm across demographic variables (e.g., age, sex, and handedness) and physical characteristics (e.g., hair density and type, and skin pigmentation), future studies should consider a stratified sampling approach. Given the relevance of physiological features for fNIRS signal quality,^56^ these variables could be quantified in a screening session (for guidelines, see Ref. 57) and included in the analysis to assess their potential effects on the effectiveness of our classification approach.

One major challenge of our study design is the difficulty of enforcing unresponsiveness in healthy participants during simulated UWS conditions. In particular, the misclassification of one participant (P01) likely reflects a failure to fully comply with the instruction to ignore task cues, resulting in classifier detection of volitional mental activity. Although UWS patients, by definition, are incapable of task engagement, healthy participants may inadvertently respond. This limitation is inherent to a simulation-based paradigm. Additional vigilance checks (e.g., eye tracking or monitoring of autonomic arousal) may provide indirect evidence of engagement, but cannot reliably detect covert mental activity. As an improvement to the current protocol, future studies could incorporate explicit post-run debriefings in which participants are asked whether they responded to any cues despite instructions, allowing researchers to exclude or repeat noncompliant runs. This would increase confidence in the validity of the simulated UNRESPONSIVE condition and reduce the risk of false-positive classifications.

A key limitation of the present study lies in the assumption that the responsive and unresponsive task conditions in healthy individuals can serve as proxies for the MCS and UWS diagnostic categories. This approach makes two strongly simplifying assumptions: first, MCS and UWS are distinct and dichotomous clinical states, and second, they can be meaningfully simulated in cognitively healthy individuals upon instruction. However, DoC is increasingly understood as a spectrum of awareness^58^ that cannot be fully replicated by healthy participants. Moreover, although participants were instructed to remain unresponsive, it is impossible to verify full suppression of task engagement, and this may differ from the neurological incapacity characteristic of UWS. Thus, our simulated conditions cannot fully reproduce MCS or UWS but rather simulate the presence or absence of volitional command following on an individual level. This study should therefore be understood as a proof-of-principle of the classification method under controlled conditions, which provides a foundation for future validation studies in actual DoC patient populations.

Future validation efforts should include testing our diagnostic tool with DoC patients. Concretely, we propose a validation study involving patients formally diagnosed with UWS and MCS using the current gold-standard behavioral assessments such as the Coma Recovery Scale-Revised (CRS-R). Patients would perform only the two RESPONSIVE runs of our individualized mental-imagery paradigm in its current form, focusing on whether the classifier can detect volitional imagery at the individual level. Patient outcomes should then be compared with behavioral assessments and, where available, EEG/fMRI indicators of covert awareness. The evaluation of classification sensitivity and specificity in a real-world clinical population and the examination of feasibility in terms of recording time, patient tolerance, and processing speed are important next steps for translating our proof-of-principle into a diagnostic tool for bedside use.

## Supplementary Material

10.1117/1.NPh.12.4.045001.s01

## Data Availability

Data will be made available upon reasonable request.

## References

[1] Laureys S. et al., “Unresponsive wakefulness syndrome: a new name for the vegetative state or apallic syndrome,” BMC Med. 8, 68 (2010). 10.1186/1741-7015-8-68 21040571 PMC2987895

[2] Edlow B. L. et al., “Recovery from disorders of consciousness: mechanisms, prognosis and emerging therapies,” Nat. Rev. Neurol. 17, 135–156 (2021). 10.1038/s41582-020-00428-x 33318675 PMC7734616

[3] Thibaut A. et al., “Therapeutic interventions in patients with prolonged disorders of consciousness,” Lancet Neurol. 18, 600–614 (2019). 10.1016/S1474-4422(19)30031-6 31003899

[4] Kondziella D. et al., “European Academy of Neurology guideline on the diagnosis of coma and other disorders of consciousness,” Eur. J. Neurol. 27, 741–756 (2020). 10.1111/ene.14151 32090418

[5] Giacino J. T. Kalmar K. Whyte J., “The JFK Coma Recovery Scale-Revised: measurement characteristics and diagnostic utility,” Arch. Phys. Med. Rehabil. 85, 2020–2029 (2004). 15605342 10.1016/j.apmr.2004.02.033

[6] Teasdale G. Jennett B., “Assessment of coma and impaired consciousness. A practical scale,” Lancet Lond. Engl. 304, 81–84 (1974). 10.1016/S0140-6736(74)91639-0 4136544

[7] Schnakers C. et al., “Diagnostic accuracy of the vegetative and minimally conscious state: clinical consensus versus standardized neurobehavioral assessment,” BMC Neurol. 9, 35 (2009). 10.1186/1471-2377-9-35 19622138 PMC2718857

[8] Coleman M. R. et al., “Towards the routine use of brain imaging to aid the clinical diagnosis of disorders of consciousness,” Brain J. Neurol. 132, 2541–2552 (2009). 10.1093/brain/awp183 19710182

[9] Schiff N. D. et al., “Brain–computer interfaces for communication in patients with disorders of consciousness: a gap analysis and scientific roadmap,” Neurocrit. Care 41, 129–145 (2024). 10.1007/s12028-023-01924-w 38286946 PMC11284251

[10] Andrews K. et al., “Misdiagnosis of the vegetative state: retrospective study in a rehabilitation unit,” BMJ 313, 13–16 (1996). 10.1136/bmj.313.7048.13 8664760 PMC2351462

[11] Childs N. L. Mercer W. N. Childs H. W., “Accuracy of diagnosis of persistent vegetative state,” Neurology 43, 1465–1465 (1993). NEURAI 0028-3878 10.1212/WNL.43.8.1465 8350997

[12] Bardin J. C. Schiff N. D. Voss H. U., “Pattern classification of volitional functional magnetic resonance imaging responses in patients with severe brain injury,” Arch. Neurol. 69, 176–181 (2012). 10.1001/archneurol.2011.892 22332186 PMC11737287

[r13] Bardin J. C. et al., “Dissociations between behavioural and functional magnetic resonance imaging-based evaluations of cognitive function after brain injury,” Brain 134, 769–782 (2011). BRAIAK 0006-8950 10.1093/brain/awr005 21354974 PMC3044833

[r14] Claassen J. et al., “Detection of brain activation in unresponsive patients with acute brain injury,” N. Engl. J. Med. 380, 2497–2505 (2019). NEJMAG 0028-4793 10.1056/NEJMoa1812757 31242361

[r15] Cruse D. et al., “Bedside detection of awareness in the vegetative state: a cohort study,” Lancet 378, 2088–2094 (2011). 22078855 10.1016/S0140-6736(11)61224-5

[r16] Galiotta V. et al., “EEG-based brain-computer interfaces for people with disorders of consciousness: features and applications. A systematic review,” Front. Hum. Neurosci. 16, 1040816 (2022). 10.3389/fnhum.2022.1040816 36545350 PMC9760911

[r17] Gibson R. M. et al., “Multiple tasks and neuroimaging modalities increase the likelihood of detecting covert awareness in patients with disorders of consciousness,” Front. Hum. Neurosci. 8, 950 (2014). 10.3389/fnhum.2014.00950 25505400 PMC4244609

[r18] Luauté J. Morlet D. Mattout J., “BCI in patients with disorders of consciousness: clinical perspectives,” Ann. Phys. Rehabil. Med. 58, 29–34 (2015). 10.1016/j.rehab.2014.09.015 25616606

[r19] Monti M. M. et al., “Willful modulation of brain activity in disorders of consciousness,” N. Engl. J. Med. 362, 579–589 (2010). NEJMAG 0028-4793 10.1056/NEJMoa0905370 20130250

[20] Owen A. M. et al., “Detecting awareness in the vegetative state,” Science 313, 1402–1402 (2006). SCIEAS 0036-8075 10.1126/science.1130197 16959998

[21] Abdalmalak A. et al., “The potential role of fNIRS in evaluating levels of consciousness,” Front. Hum. Neurosci. 15, 703405 (2021). 10.3389/fnhum.2021.703405 34305558 PMC8296905

[22] Villringer A. et al., “Near infrared spectroscopy (NIRS): a new tool to study hemodynamic changes during activation of brain function in human adults,” Neurosci. Lett. 154, 101–104 (1993). NELED5 0304-3940 10.1016/0304-3940(93)90181-J 8361619

[23] Klein F. et al., “From lab to life: challenges and perspectives of fNIRS for haemodynamic-based neurofeedback in real-world environments,” Philos. Trans. R. Soc. Lond. B. Biol. Sci. 379, 20230087 (2024). PTRBAE 0962-8436 10.1098/rstb.2023.0087 39428887 PMC11513164

[24] Benitez-Andonegui A. et al., “Guiding functional near-infrared spectroscopy optode-layout design using individual (f)MRI data: effects on signal strength,” Neurophotonics 8, 025012 (2021). 10.1117/1.NPh.8.2.025012 34155480 PMC8211086

[25] Liu Y. et al., “Detecting residual brain networks in disorders of consciousness: a resting-state fNIRS study,” Brain Res. 1798, 148162 (2023). BRREAP 0006-8993 10.1016/j.brainres.2022.148162 36375509

[26] Shu Z. et al., “fNIRS-based functional connectivity signifies recovery in patients with disorders of consciousness after DBS treatment,” Clin. Neurophysiol. Off. J. Int. Fed. Clin. Neurophysiol. 147, 60–68 (2023). 10.1016/j.clinph.2022.12.011 36702043

[27] Lu H. et al., “A functional near-infrared spectroscopy study on hemodynamic changes of patients with prolonged disorders of consciousness responding to different auditory stimuli,” BMC Neurol. 23, 242 (2023). 10.1186/s12883-023-03292-6 37353754 PMC10288743

[28] Molteni E. et al., “Bedside assessment of residual functional activation in minimally conscious state using NIRS and general linear models,” in Annu. Int. Conf. IEEE Eng. Med. Biol. Soc., pp. 3551–3554 (2013). 10.1109/EMBC.2013.6610309 24110496

[r29] Abdalmalak A. et al., “Assessing time-resolved fNIRS for brain-computer interface applications of mental communication,” Front. Neurosci. 14, 105 (2020).1662-453X 10.3389/fnins.2020.00105 32132894 PMC7040089

[r30] Kazazian K. et al., “Functional near-infrared spectroscopy: a novel tool for detecting consciousness after acute severe brain injury,” Proc. Natl. Acad. Sci. 121, e2402723121 (2024). 10.1073/pnas.2402723121 39186658 PMC11388405

[r31] Kempny A. M. et al., “Functional near infrared spectroscopy as a probe of brain function in people with prolonged disorders of consciousness,” NeuroImage Clin. 12, 312–319 (2016). 10.1016/j.nicl.2016.07.013 27547728 PMC4983150

[32] Si J. et al., “Evaluation of residual cognition in patients with disorders of consciousness based on functional near-infrared spectroscopy,” Neurophotonics 10, 025003 (2023). 10.1117/1.NPh.10.2.025003 37064779 PMC10091901

[33] Vorreuther A. et al., “It takes two (seconds): decreasing encoding time for two-choice functional near-infrared spectroscopy brain–computer interface communication,” Neurophotonics 10, 045005 (2023). 10.1117/1.NPh.10.4.045005 37928600 PMC10620514

[34] Holper L. Biallas M. Wolf M., “Task complexity relates to activation of cortical motor areas during uni- and bimanual performance: a functional NIRS study,” NeuroImage 46, 1105–1113 (2009). NEIMEF 1053-8119 10.1016/j.neuroimage.2009.03.027 19306929

[35] Naseer N. Hong K.-S., “fNIRS-based brain-computer interfaces: a review,” Front. Hum. Neurosci. 9, 3 (2015). 10.3389/fnhum.2015.00003 25674060 PMC4309034

[36] Gramfort A. et al., “MEG and EEG data analysis with MNE-Python,” Front. Neurosci. 7, 267 (2013).1662-453X 10.3389/fnins.2013.00267 24431986 PMC3872725

[37] Scholkmann F. Metz A. J. Wolf M., “Measuring tissue hemodynamics and oxygenation by continuous-wave functional near-infrared spectroscopy—how robust are the different calculation methods against movement artifacts?” Physiol. Meas. 35, 717–734 (2014). PMEAE3 0967-3334 10.1088/0967-3334/35/4/717 24622337

[38] Saager R. B. Berger A. J., “Direct characterization and removal of interfering absorption trends in two-layer turbid media,” J. Opt. Soc. Amer. A Opt. Image Sci. Vis. 22, 1874–1882 (2005). 10.1364/JOSAA.22.001874 16211814

[39] Fabbri F. et al., “Optical measurements of absorption changes in two-layered diffusive media,” Phys. Med. Biol. 49, 1183–1201 (2004). PHMBA7 0031-9155 10.1088/0031-9155/49/7/007 15128197

[40] Fuertinger S. Shapcott K. Schmiedt J. T., “ACME: asynchronous computing made ESI,” Version 2025.6, Ernst Strüngmann Institute for Neuroscience in cooperation with the Max Planck Society, https://github.com/esi-neuroscience/acme (2025).

[41] Näher T. et al., “Riemannian geometry for the classification of brain states with fNIRS,” 2024.09.06.611347. 10.1101/2024.09.06.611347 (2024).

[42] Barachant A. et al., “Classification of covariance matrices using a Riemannian-based kernel for BCI applications,” Neurocomputing 112, 172–178 (2013). NRCGEO 0925-2312 10.1016/j.neucom.2012.12.039

[43] Barachant A. Congedo M., “A plug&play P300 BCI using information geometry,” 10.48550/arXiv.1409.0107 (2014).

[44] Rivet B. et al., “xDAWN algorithm to enhance evoked potentials: application to brain-computer interface,” IEEE Trans. Biomed. Eng. 56, 2035–2043 (2009). IEBEAX 0018-9294 10.1109/TBME.2009.2012869 19174332

[45] Rivet B. et al., “Theoretical analysis of xDAWN algorithm: application to an efficient sensor selection in a P300 BCI,” in EUSIPCO 2011 - 19th Eur. Signal Process. Conf., Barcelone, Spain, pp. 1382–1386 (2011).

[46] Chen Y. et al., “Shrinkage algorithms for MMSE covariance estimation,” arXiv:0907.4698 (2009).

[47] Barachant A. et al., “Multiclass brain–computer interface classification by Riemannian geometry,” IEEE Trans. Biomed. Eng. 59, 920–928 (2012). IEBEAX 0018-9294 10.1109/TBME.2011.2172210 22010143

[48] Izzuddin T. A. Safri N. M. Othman M. A., “Mental imagery classification using one-dimensional convolutional neural network for target selection in single-channel BCI-controlled mobile robot,” Neural Comput. Appl. 33, 6233–6246 (2021). 10.1007/s00521-020-05393-6

[49] Combrisson E. Jerbi K., “Exceeding chance level by chance: the caveat of theoretical chance levels in brain signal classification and statistical assessment of decoding accuracy,” J. Neurosci. Methods 250, 126–136 (2015). JNMEDT 0165-0270 10.1016/j.jneumeth.2015.01.010 25596422

[50] Fernández-Espejo D. Norton L. Owen A. M., “The clinical utility of fMRI for identifying covert awareness in the vegetative state: a comparison of sensitivity between 3T and 1.5T,” PLOS One 9(4), e95082 (2014). POLNCL 1932-6203 10.1371/journal.pone.0095082 24733575 PMC3986373

[51] Irimia A. Horn J. D. V., “Functional neuroimaging of traumatic brain injury: advances and clinical utility,” Neuropsychiatr. Dis. Treat. 11, 2355 (2015). 10.2147/NDT.S79174 26396520 PMC4576900

[52] Li M. et al., “Detecting residual awareness in patients with prolonged disorders of consciousness: an fNIRS study,” Front. Neurol. 12, 618055 (2021). 10.3389/fneur.2021.618055 34393964 PMC8355369

[53] van Baal P. Morton A. Severens J. L., “Health care input constraints and cost effectiveness analysis decision rules,” Soc. Sci. Med.-1982 200, 59 (2018). 10.1016/j.socscimed.2018.01.026 PMC5906649 29421472

[54] Freedman S. et al., “Docs with their eyes on the clock? The effect of time pressures on primary care productivity,” J. Health Econ. 77, 102442 (2021). 10.1016/j.jhealeco.2021.102442 33684849 PMC8122046

[55] Kurz E.-M. et al., “Towards using fNIRS recordings of mental arithmetic for the detection of residual cognitive activity in patients with disorders of consciousness (DOC),” Brain Cogn. 125, 78–87 (2018). 10.1016/j.bandc.2018.06.002 29909026

[56] Wassenaar E. B. Van den Brand J. G. H., “Reliability of near-infrared spectroscopy in people with dark skin pigmentation,” J. Clin. Monit. Comput. 19, 195–199 (2005). 10.1007/s10877-005-1655-0 16244841

[57] Yücel M. A. et al., “Quantifying the impact of hair and skin characteristics on signal quality with practical recommendations for improvement,” bioRxiv 2024.10.28.620644 (2024).

[58] Giacino J. T. et al., “Disorders of consciousness after acquired brain injury: the state of the science,” Nat. Rev. Neurol. 10, 99–114 (2014). 10.1038/nrneurol.2013.279 24468878

