# Supplementary material for: Sensitive and specific fNIRS-based approach for awareness detection in disorders of consciousness: proof of principle in healthy adults
Source: Neurophotonics. 2025 Oct 15;12(4):045001. doi: 10.1117/1.NPh.12.4.045001 (PMC12521986; doi:10.1117/1.NPh.12.4.045001)
Supplement: Supplementary file 1 [file NPh_012_045001_SD001.pdf]

Supplementary Information

A Sensitive and Specific fNIRS-based Approach for Awareness Detection in Disorders of Consciousness: Proof of Principle in Healthy Adults

Lisa Bastian, Tim Näher, Anna Vorreuther, Michael Lührs, Amaia Benitez Andonegui, Pascal Fries, Lars Riecke, Bettina Sorger

This PDF includes:

Supplementary Figure S1

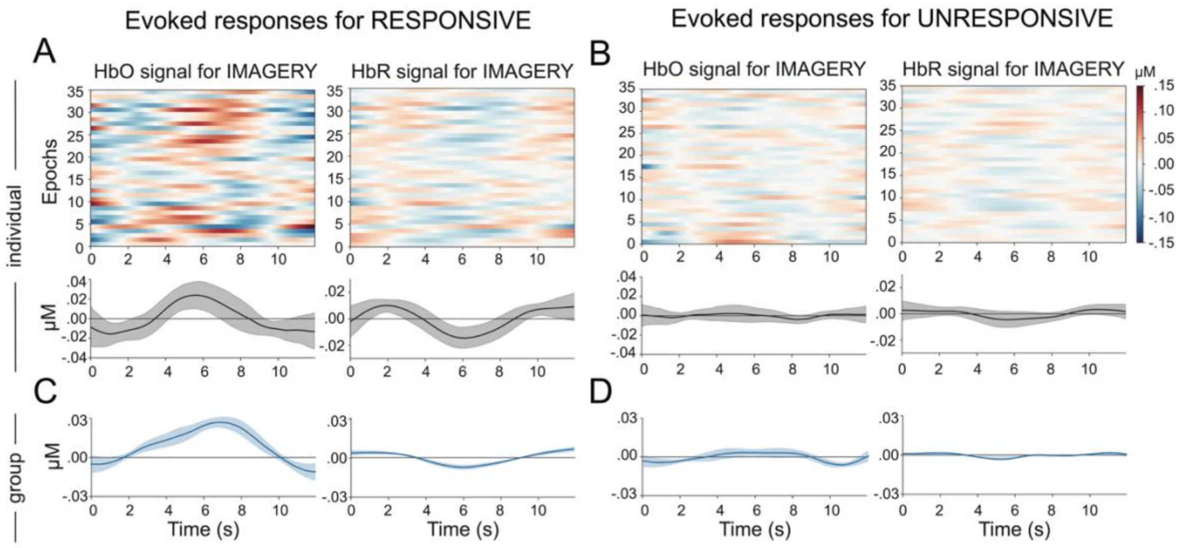

Figure S1. Single-trial hemodynamic response amplitudes and response average. A. Evoked responses over all fNIRS channels for an example participant (P02) during the RESPONSIVE runs for IMAGERY trials for HbO (left) and HbR (right). The top plot shows individual epochs on the y-axis, and the response amplitude is color-coded. The bottom plot shows the average response amplitude (shading: standard error) over epochs. **B.** Same as **A** but for the UNRESPONSIVE runs. **C.** Average response amplitude (shading: standard error) across participants for imagery and no-imagery trials during RESPONSIVE runs. **D.** Same as **C** but for UNRESPONSIVE runs.
